# An efficient fat suppression technique for stimulated-echo based CMR

**DOI:** 10.1186/1532-429X-18-S1-W6

**Published:** 2016-01-27

**Authors:** Ahmed S Fahmy, El-Sayed H Ibrahim, Nael F Osman

**Affiliations:** 1 University of Michigan, Ann Arbor, MI USA; 2 Nile University, Cairo, Egypt; 3 Johns Hopkins University, Baltimore, MD USA

## Background

Stimulated-echo acquisition mode (STEAM) is one of the key pulse sequences in CMR imaging, as it alleviates rapid magnetization decay with T2 relaxation. Fat suppression is frequently implemented in cardiac imaging to improve visualization and tissue characterization, albeit at the cost of reduced temporal resolution and signal-to-noise ratio (SNR), as well as possible increase of specific absorption rate (SAR). The purpose of this work is to develop an efficient fat suppression method (Spectrally-Presaturated Modulation (SPM)) for STEAM sequences to enable imaging at high temporal-resolution, with high SNR, and no increase in scan time.

## Methods

**Figure 1:**
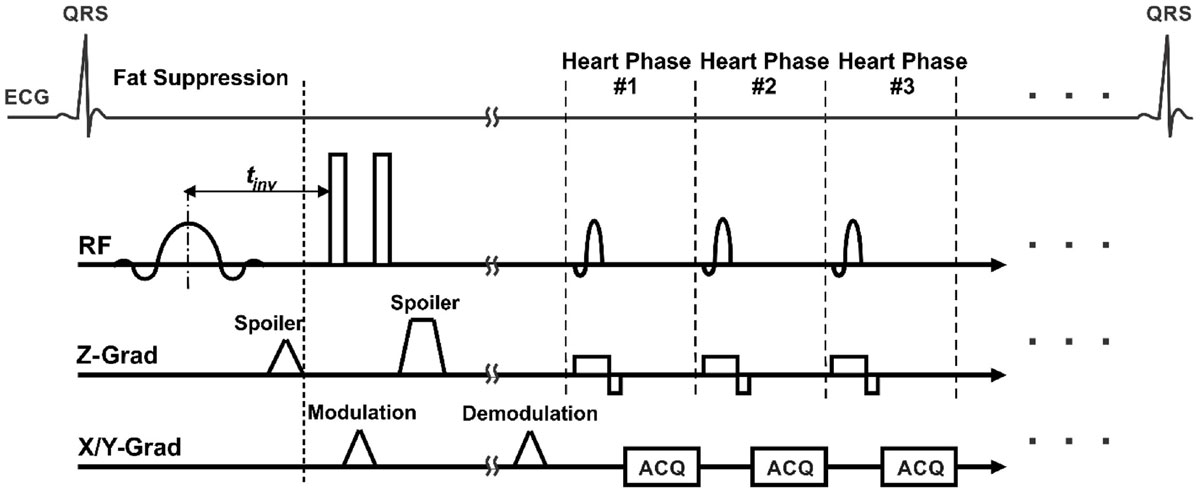
**The pulse sequence of the proposed SPM fat suppression technique**. Fat suppression takes place before the modulation stage. Therefore, only the water magnetization is modulated, which is then recalled during demodulation. Consequently, there is no need to repeat the fat suppression step during individual heart phases, which results in improved temporal resolution.

The developed method is based on saturating the fat magnetization prior to applying the STEAM modulation; therefore, only the water-content of the tissues is modulated by the sequence, resulting in fat-suppressed images without the need to run the fat suppression module during image acquisition (Figure 1). The potential significance of the proposed method has been tested in two STEAM-based cardiac MRI applications: complementary spatial-modulation of magnetization (CSPAMM) tagging, and black-blood cine imaging. In vivo experiments were conducted to evaluate the developed technique and compare it to the commonly implemented chemical-shift selective (CHESS) and water-excitation using spectral-spatial selective pulses (SSSP) fat suppression techniques.

## Results

**Figure 2:**
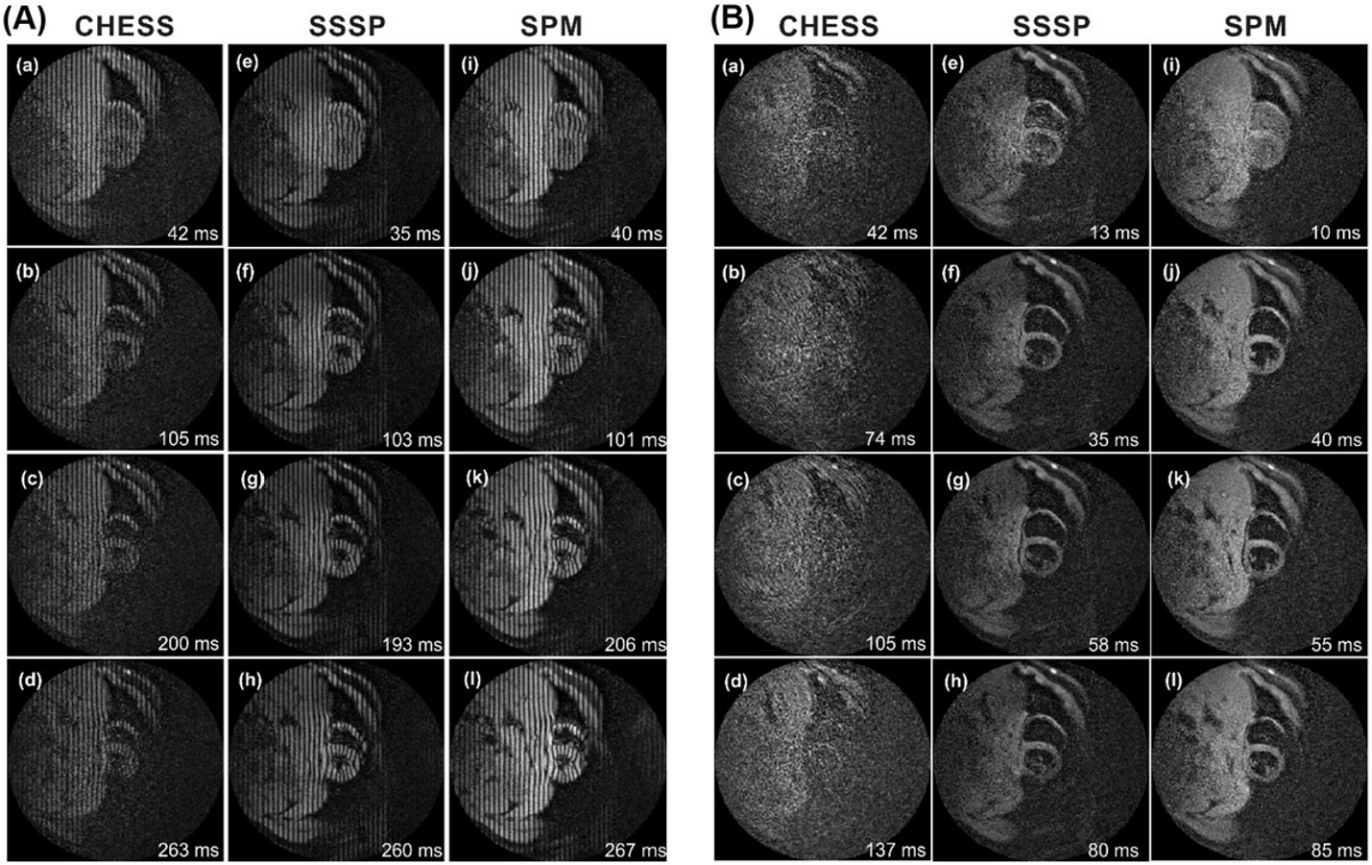
**In vivo short-axis (A) complementary spatial modulation of magnetization (SCPAMM) tagged and (B) black-blood stimulated-echo acquisition more (STEAM) images with different fat suppression technique: chemical-shift selective (CHESS), water-excitation using spectral-spatial selective pulses (SSSP), and spectrally presaturated modulation (SPM)**. The SPM images show higher signal (all images are shown with the same window and contrast values) and improved temporal resolution (large number of heart phases per cardiac cycle) compared to other fat sat techniques.

The results from the in vivo experiments showed superior performance of the proposed SPM method compared to the CHESS and SSSP techniques (Figure 2). The SPM technique provided the highest temporal resolution and SNR, as well as reduced SAR, among the studied fat suppression techniques. Moreover, the SNR did not drop with time as it did in the SSSP and CHESS techniques. The SPM technique allowed for acquiring 40 cardiac phases compared to 19 and 28 phases for the CHESS and SSSP techniques, respectively. The resulting SAR level was 0.2, 0.37, and 0.15 W/kg for the SPM, CHESS, and SSSP techniques, respectively. It can be seen that the CHESS technique suppressed the myocardial signal along with the fat signal, which could be attributed to magnetic field inhomogeneity around the heart. Although the same shimming settings were used for the SPM technique, it did not show such an artifact.

## Conclusions

An efficient technique has been presented for suppressing the fat signal in a number of STEAM-based cardiac imaging techniques. The proposed technique improves the temporal resolution of the acquired images without sacrificing SNR, SAR, or scan time, which could result in accurate parameter measurement and improved cardiac image analysis.

